# Climate Change vs. Human Activities: Conflicting Future Impacts on a High-Altitude Endangered Snake (*Thermophis baileyi*)

**DOI:** 10.3390/biology14111531

**Published:** 2025-10-31

**Authors:** Yuxue Pan, Ruiying Han, Fengbin Dai, Yu Liu, Tianjian Song, Yueheng Ren, Song Huang, Jiang Chang

**Affiliations:** 1 State Key Laboratory of Environmental Criteria and Risk Assessment, Chinese Research Academy of Environmental Sciences, Beijing 100012, China; 13520199856@163.com (Y.P.);; 2 College of Life Sciences, Anhui Normal University, Wuhu 241000, China

**Keywords:** global warming, landscape change, Tibetan hot-spring snake, species distribution modeling, Tibetan Plateau

## Abstract

**Simple Summary:**

As a high-altitude distributed reptile endemic to the Tibetan Plateau, the Tibetan hot-spring snake (*Thermophis baileyi*) is sensitive to accelerating climate change and expanding human land use, making it an ideal species for studying the effects of global changes on high-altitude ectotherms. In this study, based on field survey data and species occurrence records, we used species distribution model (SDM) under multiple future scenarios to assess the individual and combined impacts of climate and land cover change on its habitat. We identified four key environmental factors shaping its distribution and tracked changes in high-suitability habitat (HSH). The results demonstrated that anthropogenic landscape change would cause a reduction in the HSH area. Conversely, appropriate climate warming (SSP1-2.6, SSP2-4.5, SSP3-7.0) would expand the HSH area; however, this expansion effect would be diminished when superimposed with anthropogenic landscape changes. In addition, extreme warming (exceeding SSP5-8.5) would surpass the ecological niche limits of *T. baileyi*, subsequently reducing its HSH and triggering a northward shift in its distribution centroid. The quantification of climate–landscape change impacts on *T. baileyi* offers critical insights for high-altitude ectotherm distributions under global changes and evidence-based conservation planning.

**Abstract:**

Endemic ectotherms in high-altitude regions face dual threats from climate change and human activities, yet quantifiable indicators to disentangle these stressors remain limited. We developed a novel multi-scenario framework to disentangle the independent and synergistic impacts of climate change and anthropogenic landscape change on the habitat suitability of the Tibetan hot-spring snake (*Thermophis baileyi*) across the Tibetan Plateau. Our analysis was based on field survey data and species occurrence records, utilizing the species distribution model and the CA–Markov model. We identified temperature seasonality (41.8% contribution) as the primary environmental factor influencing its distribution, followed by precipitation of the coldest quarter (15.1%) and land cover (13.8%). The results showed that moderate climate warming would benefit the survival of the species, with a 24.03–38.55% gain in high-suitability habitat (HSH) area under climate change-only scenarios. However, extreme warming (exceeding SSP5-8.5) would surpass the thermal tolerance threshold of *T. baileyi*, reducing its HSH and triggering a northward shift in its distribution centroid. Landscape change reduced the HSH (5.98% reduction under land cover change-only scenario), and attenuated climate-driven gains by 4.99–11.31% under combined climate–landscape change scenarios. In addition, only one-fifth of the current HSH was covered by national natural reserves. Synergistic anthropogenic pressures critically offset climate benefits, demonstrating the need for integrated conservation strategies to address the challenges posed by both extreme climate warming and land cover change threats to mitigate future habitat degradation. The quantification of climate–land cover change impacts on *T. baileyi* offers critical insights for high-altitude ectotherm distributions under global changes and evidence-based conservation planning.

## 1. Introduction

During recent decades, the pace of global environmental change, including climate change and land cover transformation, has been unprecedented in recorded human history [1]. Global change has already damaged natural habitats so much that many terrestrial wildlife species are doomed to extinction, with many predicted to be lost within decades [2]. With global warming and the expansion of human activities, the natural habitats of many species are being damaged, leading to changes in species distribution ranges and adjustments in ecological niches [3,4,5]. Land cover change has been consistently identified as a dominant threat to biodiversity, causing substantial declines in species richness and abundance among most terrestrial species [6]. For instance, from 1995 to 2022, land use change committed 1.5% of global species to extinction [7]. These threats are particularly synergistic for ectothermic animals, as both climate and land use change erode the microhabitat heterogeneity necessary for behavioral thermoregulation [8,9]. Consequently, the interplay of shifting thermal climates and fragmented landscapes poses a fundamental challenge to their persistence, pushing many species toward their physiological limits [10,11].

The genus *Thermophis* is a relict group occurring at elevations over 3500 m [12]. It comprises three closely related species: *Thermophis baileyi* [13], the Tibetan hot-spring snake; *Thermophis zhaoermii* [14], the Sichuan hot-spring snake; and *Thermophis shangrila* [15], the Shangri-La hot-spring snake. These three species are tightly linked to high-elevation geothermal sites [16]. This dependency, however, makes them highly vulnerable to habitat destruction from the development of geothermal power stations and other commercial infrastructure [17,18].

*T. baileyi*, the Tibetan hot-spring snake (Serpentes, Colubridae), is an ectothermic species uniquely adapted to high-altitude environments, and exhibits high sensitivity to habitat alterations [19]. This nonvenomous snake is endemic to the Tibetan Plateau [20], a region with relatively extreme environments including extreme temperature fluctuations, low temperatures, high elevations, oxidative stress, intense ultraviolet radiation, and abundant geothermal resources [21]. These snakes are confined to habitats with hot springs, with the highest documented altitudinal distributions (ranging from 3600 m to 4900 m above sea level) among snakes [22]. Currently, *T. baileyi* is listed as “Endangered (EN)” by the IUCN Red List [23] and has experienced a significant change from its previous “Vulnerable (VU)” status [24] over a decade ago. Due to its strict ecological requirements and restriction to the climatically vulnerable Tibetan Plateau, *T. baileyi* is sensitive to the impacts of climate and landscape change [18], making it an ideal species for studying the effects of global changes on species distributions, especially those of ectothermic animals.

Expanding commercialization of geothermal energy has caused substantial habitat destruction, posing escalating threats to *T. baileyi* populations [18,20]. Previous research on *T. baileyi* has largely focused on taxonomic and genetic conditions, revealing mechanisms such as cold acclimation and high-elevation acclimatization [16,19]. Genomic studies have further elucidated intraspecific divergence and selection pressures in this lineage [25], while population genetic work has inferred historical refugia and current diversification drivers [20]. However, ecological aspects, particularly those related to distribution dynamics, habitat change, conservation status, and responses to anthropogenic pressure, remain poorly understood.

Given that climate and landscape change can critically impact ectothermic species by altering their thermal environments and restricting microhabitat availability [26,27,28], with the degradation of geothermal habitats and shifting climate regimes hampering both behavioural thermoregulation and dispersal [29], we hypothesized that the synergistic pressures of climate warming and landscape change were primary limiting factors for *T. baileyi*, a high-elevation ectotherm with strict microhabitat preferences tied to hot-spring resources on the Tibetan Plateau.

In this study, we aimed to address key questions regarding the conservation status, population abundance, and environmentally dependent distribution patterns of the endangered *T. baileyi* under multiple climate and landscape change scenarios across the Tibetan Plateau. By integrating species distribution modeling (SDM) with future scenario analysis, we sought to quantify the relative contributions of bioclimatic factors, landscape change, and their interactive effects on the spatial shifts of this high-altitude snake. Our findings provide the first empirical estimate of its current and future spatial distribution and demonstrate how future climate change and landscape change will shape its distribution patterns, thereby offering a scientific basis for formulating targeted conservation strategies to safeguard this and other high-elevation ectotherms in an era of global environmental change.

## 2. Materials and Methods

### 2.1. Research Framework

We developed a framework to quantify the contributions of climate change and human activities to the high-altitude distribution ectothermic species *T. baileyi* (Supplemental Figure S1). The framework primarily comprises three steps. First, we collected and selected data of occurrence records, environmental variables, and human activity data for analysis. All datasets were subsequently processed through data cleaning, variable standardization, and spatial alignment procedures. Three future scenarios were designed for simulating the future distribution. Meanwhile, the cellular automata (CA)–Markov (CA–Markov) model was employed to predict future land cover in 2050, which was driven predominantly by human activities. The MaxEnt model for *T. baileyi* was subsequently constructed, and the accuracy of the models was evaluated. Finally, the current and future suitable habitats of the species under different scenarios were simulated. Policy recommendations were proposed on the basis of the results.

### 2.2. Species Occurrence Data

**Figure 1:**
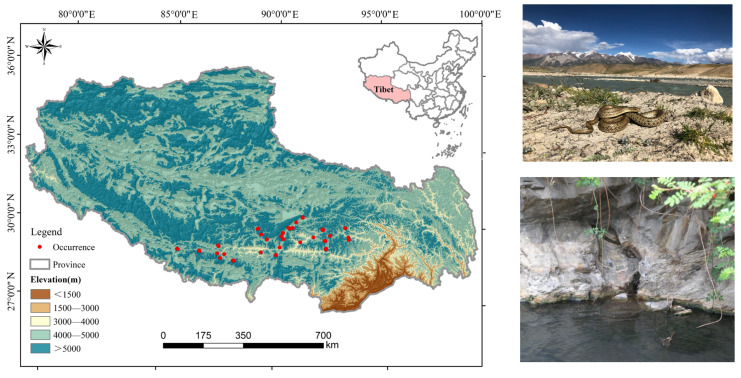
Occurrence records of *T. baileyi* used for building species distribution models.

In this study, we collected 68 occurrence records (Supplemental Table S1) of *T. baileyi,* mainly via field surveys (52 records, 149 individuals), searching the open source website Global Biodiversity Information Facility [30] (3 records) and consulting the relevant literature [17,19] (13 records) for supplementation. Duplicate records and records with obvious geocoding inaccuracies were identified and removed manually, retaining only one record per 500 m grid cell. Finally, a total of 54 records (Figure 1, Supplemental Table S2) remained for building species distribution models (SDMs) using MaxEnt version 3.6. All occurrence records were organized as “.CSV” format for analysis, as MaxEnt software requires.

Our field survey sites comprehensively covered all known distribution areas and potential microhabitats of *T. baileyi*, primarily along geothermal spring streams. Field investigations were conducted during two periods in 2019–2020 (June and August) by a total of 16 researchers organized into four teams. Field surveys of large geothermal areas employed the transect method for direct individual counts [31]. The sampling area covered no less than 5% of the distribution area. Each transect underwent four replicate surveys to ensure accuracy. For distribution points with single or few springs, a quiet stakeout approach was employed, utilizing direct counting for population assessment. Each site was surveyed for 4 consecutive days, recording the numbers of adults, subadults, and juveniles. The final count adopted the data from the day with the highest recorded numbers. Surveys were conducted around 3 p.m., when snakes are most active, even during the rainy season. Additionally, questionnaires (Supplemental Material S1) were administered to local residents to gather historical information on population trends and identify potential distribution gaps.

### 2.3. Environmental Variables

The standard 19 bioclimatic variables, key for defining species ecological niches, were used to simulate suitable habitat distributions via MaxEnt software. In addition, considering the strict microhabitat preferences tied to hot-spring resources of *T. baileyi*, land surface temperature (LST) was selected as an environmental factor, along with the other three environmental variables, land use, slope, and digital elevation model (DEM) data.

The standard 19 bioclimatic variables at a spatial resolution of 30 arc-seconds (~1 km^2^) were obtained from the WorldClim 2 database for the current and future periods [32,33]. In the WorldClim database, the current period was defined from 1970–2000. The future bioclimatic variables (averages for 2041–2060) were based on the shared socioeconomic pathway (SSP)–representative concentration pathway (RCP) combination scenario in Phase 6 of the Coupled Model Intercomparison Project (CMIP6, [34]). The climate scenarios under CMIP6 represent different socioeconomic development pathways and various trajectories of atmospheric greenhouse gas concentrations. The four scenarios SSP1-2.6, SSP2-4.5, SSP3-7.0, and SSP5-8.5 were adopted to represent the low, medium, high, and higher emission scenarios, respectively.

The LST data for 2020 was computed from Landsat 4, 5, 7, and 8 imagery using the Google Earth Engine (GEE). LST values were estimated with the Statistical Mono-Window (SMW) algorithm developed by the Climate Monitoring Satellite Application Facility (CM-SAF). The specific code and method can be found in the research of [35].

The global land cover products (GLC_FCS30) for 1990, 2005 and 2020 at 30 m using time series Landsat imagery [36] were used in this study. The GLC_FCS30 products utilize a refined classification system containing 35 land cover categories. We reclassified these 35 land cover categories into 10 categories (Supplemental Table S3).

The digital elevation model (DEM) data for 2020 was obtained from the Shuttle Radar Topography Mission (SRTM) at 90 m resolution of via the Geospatial Data Cloud site, Computer Network Information Center, Chinese Academy of Sciences (http://www.gscloud.cn). The slope data was extracted from the DEM via spatial analysis in ArcGIS 10.5.

All datasets (Table 1) were resampled to a uniform 500 m resolution using spatial analysis tools of ArcGIS 10.5 to meet MaxEnt requirements, considering a balance between computational practicality and ecological relevance. Continuous variables (e.g., bioclimatic layers) were resampled using the bilinear interpolation method, while categorical data (e.g., land cover) were processed using the majority resampling technique to preserve their discrete nature.

### 2.4. Models

#### 2.4.1. Scenario Settings

To quantify the different impacts of climate change and human activities on the distribution of *T. baileyi*, three sets of scenarios were designed in this study (Table 2).

Landscape change-only (LCO) scenario. This scenario isolated the influence of anthropogenic landscape changes on the species’ habitat distribution range while holding climate variables constant at current (2020) levels. To simulate these dynamics, the predicted 2050 land-use layer (see Section 2.4.2 for the prediction method) was input as an environmental variable to replace the 2020 land-cover layer, while the other variables remained unchanged.

Climate change-only (CCO) scenario. This scenario focused solely on the effects of climate change, incorporating four distinct Shared Socioeconomic Pathways—Representative Concentration Pathways (SSP-RCP) scenarios: SSP1-2.6, SSP2-4.5, SSP3-7.0, and SSP5-8.5. Each represented different trajectories of greenhouse gas emissions and associated climatic shifts, enabling an examination of how climate change independently affects species distributions.

Combined climate–landscape change (CCLC) scenario. This integrative scenario concurrently accounted for both landscape changes and climate change, providing a more realistic and holistic projection of the species’ potential distributional shifts under the interplay of these two major factors.

#### 2.4.2. Prediction of Future Land Cover

We used the cellular automata (CA)–Markov (CA–Markov) model [37,38] in IDRISI Terrset 17.0 to predict land cover in 2050, which served as input data for the LCO and CCLC scenarios. The CA–Markov model combines the Markov chain and CA, where the probabilities of possible land cover changes supplied by a Markov chain are spatialized by CA [39]. The model requires an initial land cover distribution and a transition matrix, based on the assumption of stationarity in the underlying drivers of land cover change [40]. This assumption is fundamental for counterfactual analysis, enabling the extrapolation of landscape dynamics under a “no intervention” hypothesis [41]. In the model, the Markov chain is used to calculate the conversion probability among land cover types, and these probabilities are subsequently used as the conversion rules of the CA to simulate the process of land cover change [41].

Land cover data for 1990, 2000, 2010 and 2020 were used to simulate future land types in 2050 with a CA–Markov model. First, land cover data for 2000 and 2010 were used to predict the land cover map in 2020. Then a validation tool within the software was used to test model accuracy by comparing our predicted 2020 land cover data with the GLC_FCS30 product for 2020, which yielded a Kappa index of 0.76, indicating a “substantial” level of accuracy and justifying the model’s use for future projection [42]. A Kappa value >0.40 is considered acceptable, and >0.75 indicates good agreement. Following successful validation, land cover data for 1990 and 2020 were used to predict the 2050 data through the previously verified model.

Driving factors representing predominant anthropogenic influences were selected. These included (1) topographic factors (DEM, slope) and (2) anthropogenic factors, including the human footprint index (HFI), human activity intensity (HAI), road network density, nighttime lights, and livestock activity intensity index. The 1 km resolution HFI for 2018 was obtained from the annual terrestrial human footprint dataset from 2000 to 2018 [43]. The HAI data for 2020 [44] was downloaded from the National Tibetan Plateau Data Center (http://data.tpdc.ac.cn). The nighttime light data for 2020 was from a prolonged artificial nighttime light dataset from China [45] downloaded from the National Tibetan Plateau Data Center. Road network data was downloaded from the Open Street Map website (http://www.openstreetmap.org/). The livestock activity intensity index for 2020 was sourced from a previous study [46]. Due to low inter-annual volatility and data availability constraints, the HFI (Human Footprint Index) for 2018 was used as a substitute for the 2020 data. All datasets (Table 1) were resampled to a uniform 500 m resolution using spatial analysis tools of ArcGIS 10.5.

#### 2.4.3. Prediction of Potential Distributions

We used the MaxEnt model, combined with environmental variables (Table 1) and occurrence records of *T. baileyi*, to simulate the current (2020) and future (2050) potential distributions of the species. MaxEnt is an effective tool developed for modelling target species’ potential geographic distributions [47] and is founded on the principle of maximum entropy from statistical mechanics and information theory [48,49]. MaxEnt has been widely applied to model the potential distribution areas of species under different climate scenarios through the analysis of current species occurrence data and environmental variables [50,51,52].

MaxEnt is capable of regulating contributions from redundant variables, rendering its robustness to the degree of predictor collinearity in model training [53]. To mitigate issues such as multicollinearity, autocorrelation, and redundancy of environmental variables that may lead to model overfitting [54], the jackknife test was used to measure the contribution rates of the remaining variables, and variables that contributed less than 1% in MaxEnt were removed [55].

In the modelling process, maximum iterations were 1000, and the number of replicates was 10. A total of 75% of the occurrence records were randomly selected for training, with the remaining 25% used for testing. The area under the curve (AUC) was used to assess the accuracy of the models. In general, the AUC value ranges from 0 to 1, and the closer the AUC is to 1, the better the model performance is. AUC values less than 0.5 indicated a failed prediction, and AUC values more than 0.9 were considered excellent [50].

Four distinct grades were categorized on the basis of the suitability index (ranging from 0 to 1) obtained from MaxEnt. The classification criteria were as follows: high-suitability habitats (HSH, *p* ≥ 0.7), medium-suitability habitats (0.35 ≤ *p* < 0.7), low-suitability habitats (0.1 ≤ *p* < 0.35), and unsuitable habitats (*p* < 0.1). Moreover, the centroid shifts of *T. baileyi* from the present to future scenarios were analysed via the zonal geometry tool in ArcGIS 10.7. The direction and distance of centroid movement of HSH were quantified.

## 3. Results

### 3.1. Dominant Environmental Variables and Response Curves

Based on the jackknife test, a total of 9 dominant factors (Table 3) were identified and retained as the environmental variables for predicting the distribution of *T. baileyi*. Among these variables, temperature seasonality (bio4) emerged as the most significant factor, contributing 41.8% to the distribution. This was followed by precipitation of the coldest quarter (bio19), with a 15.1% contribution, land cover type at 13.8%, and annual mean temperature (bio1), accounting for 10.2%. In total, these top four factors had a cumulative contribution of 80.9%, highlighting their predominant role in shaping the distribution pattern of *T. baileyi*. The identified factors collectively define the unique ecological niche of *T. baileyi*. The dominance of temperature seasonality and cold-season precipitation highlights its specific adaptation to geothermal buffering and reveals its inherent vulnerability to environmental changes that disrupt these conditions.

**Figure 2:**
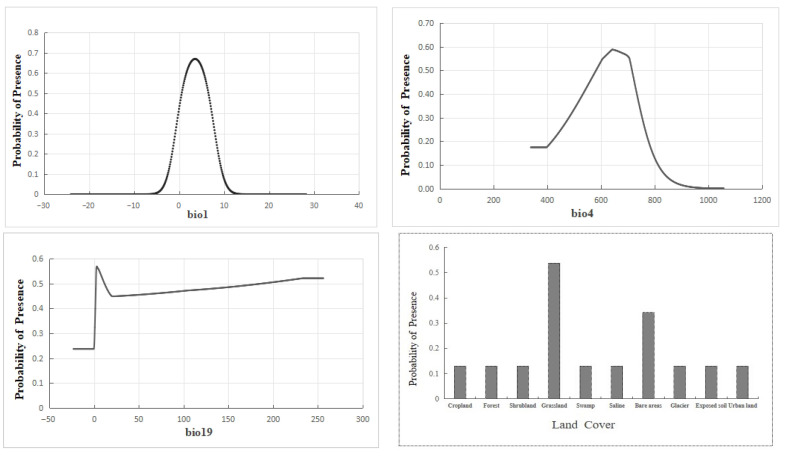
Response curves of the relationships between the distribution probability of *T. baileyi* and the dominant environmental variables.

The response curves reflect the relationships between the distribution probability and dominant environmental variables [56]. As shown in Figure 2, the presence probability of *T. baileyi* varied parabolically with bio1 and bio4. The annual mean temperature (bio1) demonstrated an optimal range of 0.62–6.50 °C, and the temperature seasonality (bio4, standard deviation ×100) showed maximal suitability within 583.26–715.59. Bare areas and sparse herbaceous areas were the land cover types most suitable for *T. baileyi* survival. The probability of *T. baileyi* increased with precipitation of the coldest quarter (bio19).

### 3.2. Comparison of Current and Future Distributions Under Different Scenarios

#### 3.2.1. Comparison of Current and Future Distributions Driven by Landscape Change

**Figure 3:**
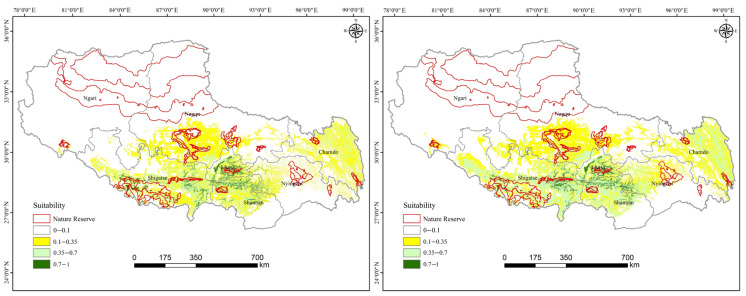
Current (**left**) and future (**right**) potentially suitable habitat distributions of *T. baileyi* under the LCO scenario.

Utilizing the MaxEnt model, the current and future suitable habitat distributions of *T. baileyi* under the LCO scenario were well simulated (Figure 3). Both training and test AUC values exceeded 0.9, indicating excellent predictive accuracy. Currently, the HSH for *T. baileyi* was predominantly clustered in areas along the middle and lower reaches of the Yarlung Zangbo River that are rich in geothermal resources, including the cities of Lhasa, Shigatse, Shannan, and Nyingchi, covering approximately 18,403.80 km^2^ (1.50% of the study area).

Under the LCO scenario, our coupled CA–Markov and MaxEnt modelling framework projected a 5.98% (978.89 km^2^) reduction in HSH by 2050, decreasing to 15,401.32 km^2^ (Table 4). This contraction was largely fueled by landscape transformations, with HSH gradually degrading and transforming into medium-suitability habitats during 2020–2050. 

**Figure 4:**
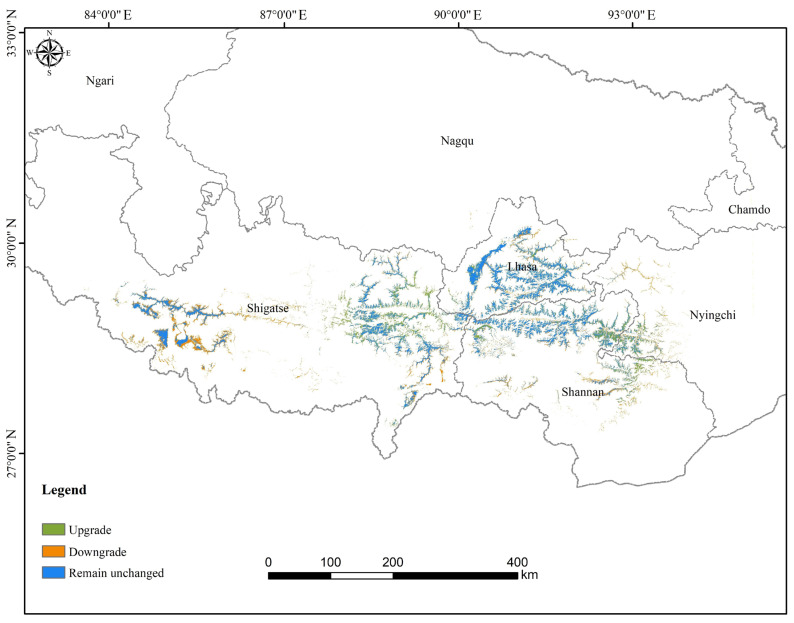
Spatial dynamics of high-suitability habitats for *T. baileyi* under the LCO scenario from 2020 to 2050.

The spatial dynamics of HSHs under the LCO scenario are shown in Figure 4. Results revealed that 34.03% of the current HSH was degraded to medium-suitability habitats, while 4.26% of medium-suitability and 0.40% of low-suitability habitats transitioned upward to HSHs. Under this scenario, 29.85% of future HSHs originated from upward transitions of medium and low-suitability areas, and the remaining 70.15% were persistent HSHs maintained from current conditions. No transitions occurred between HSHs and unsuitable habitats. Spatial patterns (Figure 4) indicate that habitat degradation was concentrated in bare rock and bare soil areas of central Shigatse, whereas habitat improvement occurred primarily in eastern Shigatse and grasslands at the border area between the Nyingchi and Shannan prefectures.

#### 3.2.2. Comparison of Current and Future Distributions Driven by Climate Change

**Figure 5:**
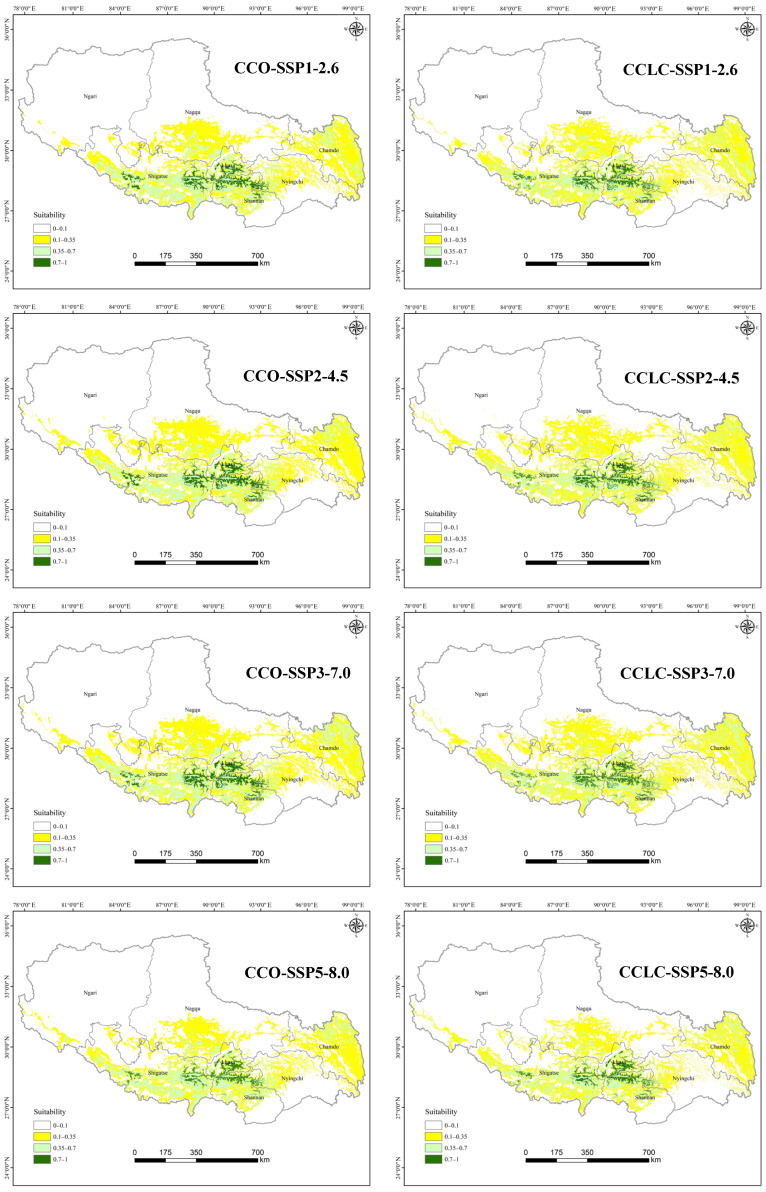
Predicted distributions of *T. baileyi* under the CCO and CCLC scenarios for the 2050s.

**Figure 6:**
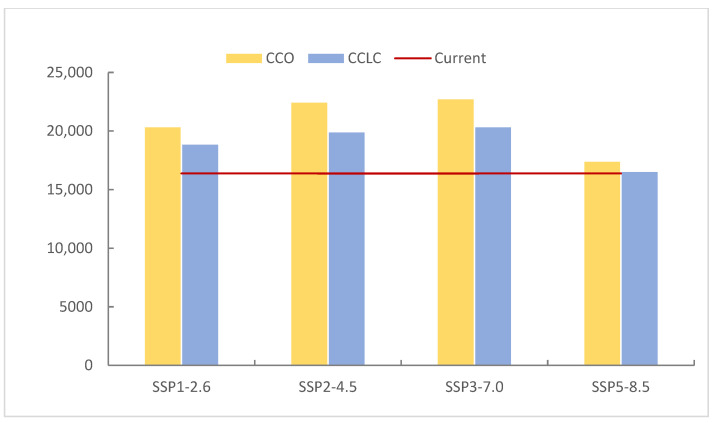
The different areas of HSH for *T. baileyi* under the current and future scenarios.

The simulated potential distributions of *T. baileyi* under four future climate change only scenarios (CCO-SSP1-2.6, CCO-SSP2-4.5, CCO-SSP3-7.0, and CCO-SSP5-8.5) for the 2050s are presented in Figure 5. Simulations revealed an expansion of HSH across all the scenarios relative to the current conditions (2020), with area increases of 24.03% (CCO-SSP1-2.6), 36.84% (CCO-SSP2-4.5), 38.55% (CCO-SSP3-7.0), and 6.00% (CCO-SSP5-8.5) (Table 4). Correlations between future emission levels and the HSH area displayed a convex trajectory, peaking in the CCO-SSP3-7.0 scenario (Figure 6). The HSH area reached its maximum extent of 22,694.66 km^2^ under the CCO-SSP3-7.0 scenario. However, under the highest emissions scenario (SSP5-8.5), the HSH growth rate drastically decelerated to 6.00% (from 38.55% under SSP3-7.0), and the predicted HSH area decreased to 17,367.78 km^2^. The medium-suitability habitats exhibited growth across all four climate change scenarios, with projected increases ranging from 2.79% to 20.47% relative to the baseline (2020) conditions. This pattern was mirrored in low-suitability habitats, which also exhibited universal gains under all scenarios, with projected increases of 5.29% to 27.30%. Conversely, unsuitable habitats exhibited an inverse pattern, with area reductions of −1.51% to −7.60% across all scenarios.

**Figure 7:**
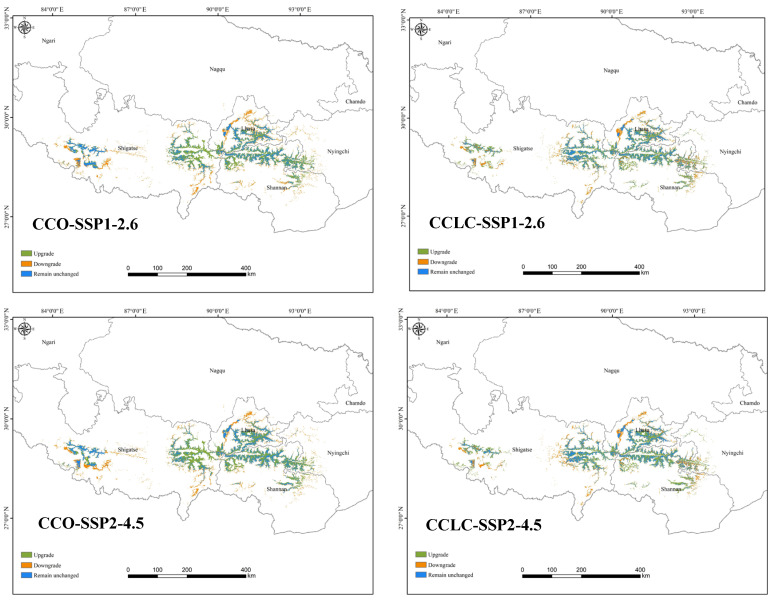

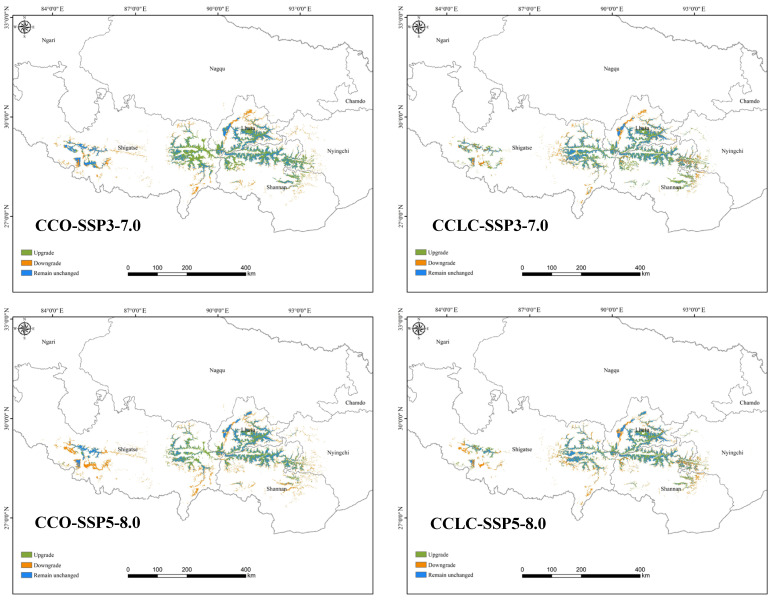
Future HSH compositions under the CCO and CCLC scenarios.

Figure 7 illustrates future HSH compositions under the four future climate change-only scenarios. Under the CCO-SSP1-2.6 scenario, 46.18% of the current HSH degraded to medium-suitability habitats, whereas 10.61% of the medium and 1.06% of the low-suitability habitats upgraded to HSH, forming 56.89% of the future HSH. Under the CCO-SSP2-4.5 scenario, 43.92% of HSH degradation was offset by 12.06% medium- and 1.19% low-suitability improvements, forming 58.47% of the future HSH and resulting in a 36.84% increase in future HSH. Under the CCO-SSP3-7.0 scenario, 43.32% of HSH loss was balanced by 12.20% of the gains in medium- and 1.22% of the gains in low-suitability habitats, forming 58.54% of the future HSH and resulting in a 38.55% increase in the future HSH. Under the CCO-SSP5-8.5 scenario, 51.44% of the current HSH degraded to lower-suitability habitats, whereas 9.06% of the medium- and 0.62% of the low-suitability habitats transitioned to HSH, forming 53.68% of the future HSH and resulting in a 6.03% increase in the future HSH. A key finding is the substantial transformation of the habitat landscape, characterized by concurrent degradation of current HSH and upgrades from lower-suitability classes. The overall increase in the total HSH area in the future scenarios masks this significant internal redistribution, indicating a future of heightened habitat turnover.

#### 3.2.3. Comparison of Current and Future Distributions Driven by Both Climate Change and Landscape Change

Under these combined CCLC scenarios, the trends in HSH area changes mirrored those under the CCO scenarios. All four future emission scenarios exhibited increases in HSH relative to the current conditions (Figure 6), with area increases of 14.90% (CCO-SSP1-2.6), 21.36% (CCO-SSP2-4.5), 24.00% (CCO-SSP3-7.0), and 0.74% (CCO-SSP5-8.5). The gained area first increased and then decreased with increasing emission intensity scenarios, peaking in the CCO-SSP3-7.0 scenario before sharply declining under the CCO-SSP5-8.5 scenario, indicating a climatic optimum threshold. The HSH area peaked at 20,311.60 km^2^ under the CCO-SSP3-7.0 scenario and then decreased to 16,500.78 km^2^ under the CCO-SSP5-8.5 scenario, which was only 120.57 km^2^ larger than the current HSH area.

Despite consistent trends in habitat expansion, the comparative analysis between climate change-only (CCO) and combined climate–land-cover change (CCLC) scenarios revealed that HSH areas under the CCLC scenarios were consistently smaller than those under the CCO scenarios across all four future climate scenarios (SSP1-2.6, SSP2-4.5, SSP3-7.0, and SSP5-8.5) (Figure 6). That is, landscape changes attenuated the climate-driven habitat gains for *T. baileyi*. The effect of landscape change reduced potential climate benefits by 5.29–15.48%, underscoring the need for integrated management policies that address both climatic and anthropogenic pressures.

Habitat suitability transitions and future HSH compositions under the CCLC scenarios were analysed (Figure 7). Under the CCLC-SSP1-2.6 scenario, 43.92% of the current HSHs degraded to lower-suitability habitats, whereas 6.69% of the medium- and 0.04% of the low-suitability habitats upgraded to HSHs, forming 53.87% of the future HSH. Under the CCLC-SSP2-4.5 scenario, 42.16% HSH degradation was offset by 7.21% medium- and 0.04% low-suitability improvements, forming 54.95% of the future HSH, resulting in a 21.36% increase in future HSH. Under the CCLC-SSP3-7.0 scenario, 40.55% of HSH loss was balanced by 7.34% medium- and 0.03% low-suitability habitat gains, forming 54.69% of the future HSH, resulting in a 24.00% increase in future HSH. Under the CCLC-SSP5-8.5 scenario, 46.77% of the current HSH degraded to lower-suitability habitats, whereas 5.46% of the medium- and 0.02% of the low-suitability habitats transitioned to HSH, forming 50.06% of the future HSH. The results imply that medium-suitability habitat contributed the most upgradation to HSH, and the transition from the low-suitability habitat to HSH remained minimal (<0.05%) across the scenarios.

### 3.3. Centroid Shifts Under Different Scenarios

**Figure 8:**
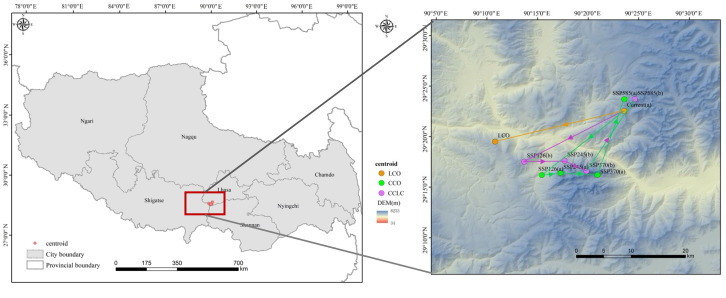
Distribution centroid shifts of *T. baileyi* under different scenarios.

The distribution centroid shift serves as a key indicator of spatiotemporal habitat redistribution for species under environmental changes. As shown in Figure 8, the current distribution centroid of the *T. baileyi* HSH was located in southwestern Lhasa city (E90.39, N29.37), with an altitude of 5167 m.

Under the LCO scenario for the 2050s, accounting for only the influence of landscape changes, the centroid shifted southwestward, with the latitude lower than the current centroid by a distance of 21.48 km. Notably, this shift was also associated with a descent of 566 m in elevation, adding a critical dimension to the habitat change.

Under the CCO-SSP1-2.6 scenario for the 2050s, the centroid shifted southwestward by a smaller distance of 17.63 km to a lower elevation of 4361 m, indicating a potential for range expansion into low-elevation areas under mild warming. As emission levels increased, the centroid generally moved eastward under the CCO-SSP2-4.5 and CCO-SSP3-7.0 scenarios, shifting 3.02 km and then 5.92 km to the eastern part of Shigatse at elevations of 4679 m and 5567 m, respectively. In contrast, under the CCO-SSP5-8.5 scenario, the centroid moved northward to higher latitudes in Lhasa city, rising to 5609 m—a pattern indicative of a forced range contraction, in which the species was squeezed into higher elevations to escape extreme heat. Overall, the centroid’s elevation rose with increasing emission levels and exceeds the current centroid elevation under the CCO-SSP3-70 scenario.

Under the CCLC scenarios for the 2050s, combining both landscape change and climate change, the trend of centroid shifts was similar to that under the CCO scenarios but with relatively higher latitudes by distances of 18.48 km, 6.46 km, 4.01 km, and 15.24 km. The centroid’s elevation followed a ‘down-then-up’ trajectory with increasing emission levels (rising from 4718 m under the CCLC-SSP1-26 scenario to 5553 m under the CCLC-SSP3-70 scenario and then down to 5326 m under the CCLC-SSP5-85 scenario) and exceeded the current centroid elevation under the CCLC-SSP3-70 scenario.

In summary, with the exception of the CCO-SSP5-8.5 and CCLC5–8.5 scenarios, under which the centroids of *T. baileyi* shifted northwards to higher latitudes, all the other scenarios exhibited a predominant southwestward migration of centroids toward lower latitudes. This latitudinal shift was paralleled by a complex elevational trajectory: initial descent under low emissions signaled potential low-elevation expansion, whereas the subsequent ascent under intermediate and high emissions—particularly exceeding the current centroid elevation under SSP3-7.0 scenarios—reflected a forced upward contraction as thermal conditions deteriorated. Furthermore, the centroids exhibited progressive eastwards shifts with increasing emission levels until they surpassed a critical climatic threshold under the SSP5-8.5 scenario, beyond which the centroids reversed in direction to migrate northwards.

## 4. Discussion

### 4.1. Effects of the Critical Environmental Variables

The distribution of *T. baileyi* is tightly regulated by multiple environmental factors. Its distribution exhibits a unimodal response to both annual mean temperature and temperature seasonality, with an optimal range within the intervals (bio1: 0.62–6.50 °C; bio4: 583.26–715.59). This pattern highlights its strong dependence on the unique thermal regime of the Tibetan Plateau. The extreme winter cold on the plateau (often below −10 °C) poses a significant physiological challenge for an ectotherm. Consequently, *T. baileyi* is obligately dependent on geothermal springs, utilizing them as critical thermal refugia to maintain body temperature and survive periods of cold stress [19]. This behavioral thermoregulation is a key ecological adaptation enabling its persistence in this high-altitude environment.

The results indicate that temperature seasonality is the most critical factor influencing the distribution of *T. baileyi*. This suggests that although the species has evolved an efficient thermal-sensing system to detect and respond to ambient temperature seasonality, the substantial contribution of this factor (41.8%) implies that any changes in temperature seasonality, such as those caused by climate change, could have a significant impact on the habitat suitability and distribution range of the species.

The second most important factor, precipitation of the coldest quarter (15.1%), highlights the dependency of *T. baileyi* on specific moisture conditions during the coldest period of the year. This could be related to the availability of prey, species thermoregulation requirements, or the need for suitable micro-habitats during this season [57]. The primary prey of *T. baileyi* include *Nanorana parkeri*, *Triplophysa*, and *Schizothorax oconnori*, which are found in streams, rivers or still ponds [58]. The precipitation of the coldest quarter significantly impacts foraging efficiency for these prey species by constraining the snake’s activity and reducing prey availability. Low temperatures and snow cover limit the thermal opportunities for *T. baileyi* to forage, while simultaneously making its prey less active and accessible, thereby creating a compound effect that severely limits successful foraging during this critical period.

Land cover (13.8%) also played a notable role in determining the distribution of the species. This may be because these snakes have a strong preference for habitats (sparse grassland and bare rock) with hot springs as an adaptive strategy to climate change [18,22]. The commercial exploitation of hot springs and conversion of natural sparse grassland not only directly eliminates habitat but also disrupts the temperature and precipitation regimes critical for *T. baileyi*. This degradation leads to smaller, more isolated sub-populations with reduced genetic exchange and increased extinction risk [59]. Thus, effective conservation must proactively regulate land-use changes in key areas to maintain landscape connectivity and habitat quality [18].

The cumulative contribution of these top four factors reaching 80.9% suggests that conservation strategies for *T. baileyi* should prioritize areas where these environmental conditions are favourable and relatively stable. It also emphasizes the need for monitoring and understanding the interactions between these factors, as well as their potential changes due to anthropogenic activities and climate change, to ensure the long-term survival and distribution of the species. Additionally, further research could be conducted to investigate the specific mechanisms through which these factors affect *T. baileyi*, such as their impact on its behavior, physiology, and ecological interactions, which would provide more insights into the species’ ecological requirements and conservation needs.

### 4.2. Changes in Suitable Habitats Under Different Scenarios

The simulated changes in habitat suitability for *T. baileyi* under future scenarios reveal critical insights into the vulnerability and adaptive capacity of this species. Across all future scenarios, whether land-use change is incorporated or excluded, the unimodal response of HSH—peaking at SSP3-7.0 before declining sharply under SSP5-8.5—suggests a specific climatic optimum under appropriate warming. This pattern aligns with the complex effects of warming on ectotherms, where initial benefits may reverse into severe stresses [60]. The sharp decline under the highest emission scenarios (CCO scenarios: from 38.55% to 6.03%; CCLC scenarios: from 24.00% to 0.74%) underscores a fundamental shift in environmental suitability, likely driven by climate variables exceeding the species’ ecological niche limits. It has been found that exposure to extremely warm conditions may compel snakes to reduce daytime activity to avoid heat stress, thereby curtailing opportunities for foraging and reproduction. This is particularly threatening to species already inhabiting colder environments [61]. This highlights the vulnerability of *T. baileyi* to extreme climate change, regardless of landscape considerations.

Although the widespread expansion of high-, medium- and low-suitability habitats across almost all scenarios that consider future climate changes may suggest short-term adaptive potential, the underlying ecological processes and long-term conservation challenges require deeper exploration. Warming may “habitatize” previously marginal cold zones, such as alleviating winter physiological stress due to rising minimum temperatures, so that some medium- and low-suitability habitats could be upgraded to HSHs in the future. Notably, the universal decrease in unsuitable area (−1.51% to −7.60%) implies that climate change is restructuring rather than simply reducing or increasing the species’ fundamental niche.

Our projections reveal that future landscape changes are expected to reduce HSH for *T. baileyi* by 4.99–11.31% compared to a climate change-only future (Table 4). The offset was most severe under the SSP2-6.5 scenario, where HSH expansion (relative to the current conditions) plunged from +36.84% (CCO) to +21.36% (CCLC). This indicates that human activities, particularly geothermal exploitation, can suppress the transition from medium-suitability habitat to HSH, thereby curtailing potential habitat gains. For conservation planning, this underscores the critical need to explicitly regulate land-use changes, especially geothermal development, within and around current high- and medium-suitability habitats to secure the species’ adaptive capacity under global environmental change. The near absence of low-to-high-suitability habitat transitions under the CCLC scenarios (all smaller than 0.05%), compared with 0.62–1.22% under the CCO scenarios, reveals a critical constraint imposed by human activities, as well as stringent requirements of *T. baileyi* for the habitat geothermal environment. Such constraints likely arise from human activities such as urbanization and road construction, which degrade habitats and fragment landscapes [62].

### 4.3. Conservation Implications

The overlap between the national nature reserves and the varying suitability levels of *T. baileyi* habitats was computed (Figure 3). The area of HSH encompassed by the national nature reserves was 3459.82 km^2^, which accounted for only 21.12% of the simulated HSH, suggesting that the most critical habitats have not been adequately protected. The protected HSHs were distributed mainly in the Black-necked Crane National Nature Reserve in the middle reaches of the Yarlung Zangbo River. The core distribution of the hot-spring snake genus (*Thermophis*) encompasses the Yarlung Zangbo Suture Zone and the central Hengduan Mountains [15,22]—a distribution tightly linked to geothermal springs, an adaptation forged by the Tibetan Plateau’s uplift [25]. Critically, all three *Thermophis* species (*T. baileyi*, *T. shangrila*, and *T. zhaoermii*) are listed as Endangered (EN) on the IUCN Red List of Threatened Species due to their documented population declines, with their occurrence in nature reserves still unconfirmed [15,20,23]. Therefore, this assessment highlights a substantial gap in the current conservation network, underscoring the imperative to re-evaluate and strategically expand protected areas to fully encompass the core geothermal habitats essential for the genus’s survival.

Our field surveys found that this snake exists amidst widespread habitat modification, as nearly all hot springs have been altered or utilized for human purposes, leading to the degradation of key nesting sites and a loss of wetland area. Critically, our study indicates that the population is likely to decline under the pressures of landscape change or extreme climate warming. Ex situ conservation has proven extremely challenging and costly; our attempts to maintain the species in captivity at Tibet University and in facilities in inland China have all ended in failure. Given the widespread habitat degradation and the infeasibility of captive breeding, we conclude that reinforcing in situ conservation is urgently needed.

## 5. Conclusions

As the highest-altitude distributed reptile distributing only in the Tibetan Plateau, China, *T. baileyi* is an ideal species for studying the effects of global changes on high-altitude ectotherms. This study presented the first quantitative estimate of the global population size and predicted the distribution range of the snake by integrating empirical field data with mechanistic habitat modeling under multiple future scenarios.

We developed a novel multi-scenario framework to disentangle the independent and synergistic impacts of climate change and landscape change on its habitat suitability. Our results indicate that strengthening the protection of critical habitats for *T. baileyi* and adjusting conservation strategies to address the challenges posed by synergistic extreme climate and land cover change threats are essential.

On the one hand, more HSHs, especially areas around critical geothermal springs (Supplemental Table S4), need to be included in nature reserves. These micro-habitats support most of breeding populations and buffer thermal extremes [22]. On the other hand, although appropriate warming (SSP1-2.6 to SSP3-7.0) would expand HSHs, extreme emissions (SSP5-8.5) would yield nearly zero net gains. This finding underscores the need for dual mitigation—curbing emissions while regulating geothermal development. In addition, to mitigate habitat fragmentation and facilitate climate-driven range shifts, we urgently propose constructing climate-adaptive wildlife corridors along the projected migration paths, incorporating underpasses (box culverts) in roadkill hotspots caused by vehicular collisions. Since local communities traditionally uphold sacred reverence and protection customs toward snakes, building artificial snake dens and implementing community co-management to buffer anthropogenic pressures would be a culturally aligned and effective conservation strategy.

A recognized limitation of this study is the potential spatial bias in our species occurrence data. Records are inevitably clustered around accessible geothermal sites and historical survey localities, which may lead to an underestimation of the species’ fundamental niche and an overestimation of habitat suitability in poorly surveyed regions. Furthermore, our projections of HSH change are sensitive to the choice of threshold, which directly influences the absolute estimates of habitat gain and loss. In addition, our predictions assume niche conservatism and disregard potential evolutionary adaptations, a limitation common to SDM approaches [63]. Furthermore, the models do not account for interspecific interactions or direct anthropogenic pressures (e.g., tourism development near hot springs). Future studies integrating physiological tolerance assays and dispersal capacity models could help to refine these predictions. A key consideration in modeling the distribution of *T. baileyi* is its reliance on geothermal hot springs. Our study used Land Surface Temperature to identify areas with a suitable thermal regime at the landscape scale, providing a robust foundation for identifying potential habitats. We acknowledge that the direct mapping of all individual spring locations through future field surveys would be a valuable next step to enhance the spatial precision of habitat predictions.

## Figures and Tables

**Table 1:** Environmental variables used to simulate the potential distributions of *T. baileyi*.

Category	Variables	Description	Unit	Source	Resolution
Bioclimatic variables	bio1	Annual mean temperature	°C	WorldClim 2 database for the current and future periods [32]	30 s
	bio2	Mean diurnal range	°C		
	bio3	Isothermality (bio2/bio7) (×100)	–		
	bio4	Temperature seasonality (standard deviation ×100)	–		
	bio5	Max temperature of the warmest month	°C		
	bio6	Min temperature of the coldest month	°C		
	bio7	Temperature annual range (Bio5-Bio6)	°C		
	bio8	The mean temperature of the wettest quarter	°C		
	bio9	The mean temperature of the driest quarter	°C		
	bio10	The mean temperature of the warmest quarter	°C		
	bio11	The mean temperature of the coldest quarter	°C		
	bio12	Annual precipitation	mm		
	bio13	Precipitation of the wettest month	mm		
	bio14	Precipitation of the driest month	mm		
	bio15	Precipitation seasonality	mm		
	bio16	Precipitation of the wettest quarter	mm		
	bio17	Precipitation of the driest quarter	mm		
	bio18	Precipitation of the warmest quarter	mm		
	bio19	Precipitation of the coldest quarter	mm		
Terrain factors	DEM	Digital Elevation Model	m	Geospatial Data Cloud	90 m
	Slope	Slope	m	Extracted from DEM	90 m
Habitat factors	Land cover	35 land-cover categories for 1990, 2000, 2010 and 2020	_	[36]	30 m
	LST	Land Surface Temperature	°C	Computed from Landsat 4, 5, 7, and 8 within Google Earth Engine (GEE) [35]	30 m

**Table 2:** Descriptions of the different scenarios.

Scenarios		Scenario Description
Landscape Change-Only Scenario	LCO	The predicted 2050 land-cover layer was imported into the simulation model to replace the 2020 land-cover layer, while the other variables remained unchanged.
Climate Change-Only (CCO) Scenario	CCO-SSP1-2.6	The future 19 bioclimate variables (averages for 2041–2060) under SSP1-2.6, SSP2-4.5, SSP3-7.0, and SSP5-8.5 scenarios were separately imported into the simulation models to replace the current 19 bioclimate variables, while the other variables remained unchanged.
	CCO-SSP2-4.5	
	CCO-SSP3-7.0	
	CCO-SSP5-8.5	
Combined Climate–Landscape Change (CCLC) Scenario	CCLC-SSP1-2.6	Both the future land-cover layer and future 19 bioclimate variables were imported to replace the corresponding current data.
	CCLC-SSP2-4.5	
	CCLC-SSP3-7.0	
	CCLC-SSP5-8.5	

**Table 3:** Dominant environmental variables for the potential distribution of *T. baileyi*.

Variable	Percent Contribution (%)	Permutation Importance
bio4	41.8	7.3
bio19	15.1	2.9
Land cover	13.8	5.4
bio1	10.2	3.2
bio12	6.2	6.3
bio11	3.8	47.1
LST	3.7	0.5
bio9	2.4	16.4
bio14	2.1	1.8

**Table 4:** Simulated areas and increase/decrease rates of different suitability levels under current and future scenarios.

Current and Future Scenarios		Simulated Areas/km2				Increase/Decrease Rates (%)			
		Unsuitable	Low Suitability	Medium Suitability	High Suitability	Unsuitable	Low Suitability	Medium Suitability	High Suitability
Current		903,712.01	191,890.35	90,217.44	16,380.21	/	/	/	/
LCO		838,683.21	197,494.50	150,620.97	15,401.32	−7.20	2.92	66.95	−5.98
CCO	SSP1-2.6	855,617.77	220,188.49	106,077.03	20,316.71	−5.32	14.75	17.58	24.03
	SSP2-4.5	835,625.62	244,272.78	99,887.64	22,413.96	−7.53	27.30	10.72	36.84
	SSP3-7.0	835,072.98	235,748.58	108,683.78	22,694.66	−7.60	22.86	20.47	38.55
	SSP580	890,052.23	202,048.28	92,731.71	17,367.78	−1.51	5.29	2.79	6.03
CCLC	SSP126	876,995.89	212,011.51	94,371.51	18,821.08	−2.96	10.49	4.60	14.90
	SSP245	858,734.02	234,776.35	88,810.78	19,878.84	−4.98	22.35	−1.56	21.36
	SSP370	855,752.27	228,920.30	97,215.83	20,311.60	−5.31	19.30	7.76	24.00
	SSP580	908,668.61	194,841.80	82,188.82	16,500.78	0.55	1.54	−8.90	0.74

## Data Availability

The occurrence records of the Tibetan hot-spring snake in the paper are detailed in Supplemental Tables S1 and S2. The database sources and the original methodological papers of all environmental variables have been cited in the paper. The study involves no data subject to privacy or ethical restrictions.

## Supplementary Materials

The following supporting information can be downloaded at: https://www.mdpi.com/article/10.3390/biology14111531/s1, Figure S1. Research framework of this study. Table S1. 68 Occurrence records of *T. baileyi.* Table S2. 54 Occurrence records of *T. baileyi.* Table S3. Land-use reclassification. Table S4. Priority Conservation Area. Material S1. Questionnaire for the Tibetan Hot-spring Snake (*Thermophis baileyi*).
