# Supplementary material for: Climate Change vs. Human Activities: Conflicting Future Impacts on a High-Altitude Endangered Snake (Thermophis baileyi)
Source: Biology (Basel). 2025 Oct 31;14(11):1531. doi: 10.3390/biology14111531 (PMC12650207; doi:10.3390/biology14111531)
Supplement: Supplementary file 1 [file biology-14-01531-s001.zip › biology-3882330-supplementary.pdf]

The supplemental file includes:

Figures S1

Tables S1-S4

Material S1

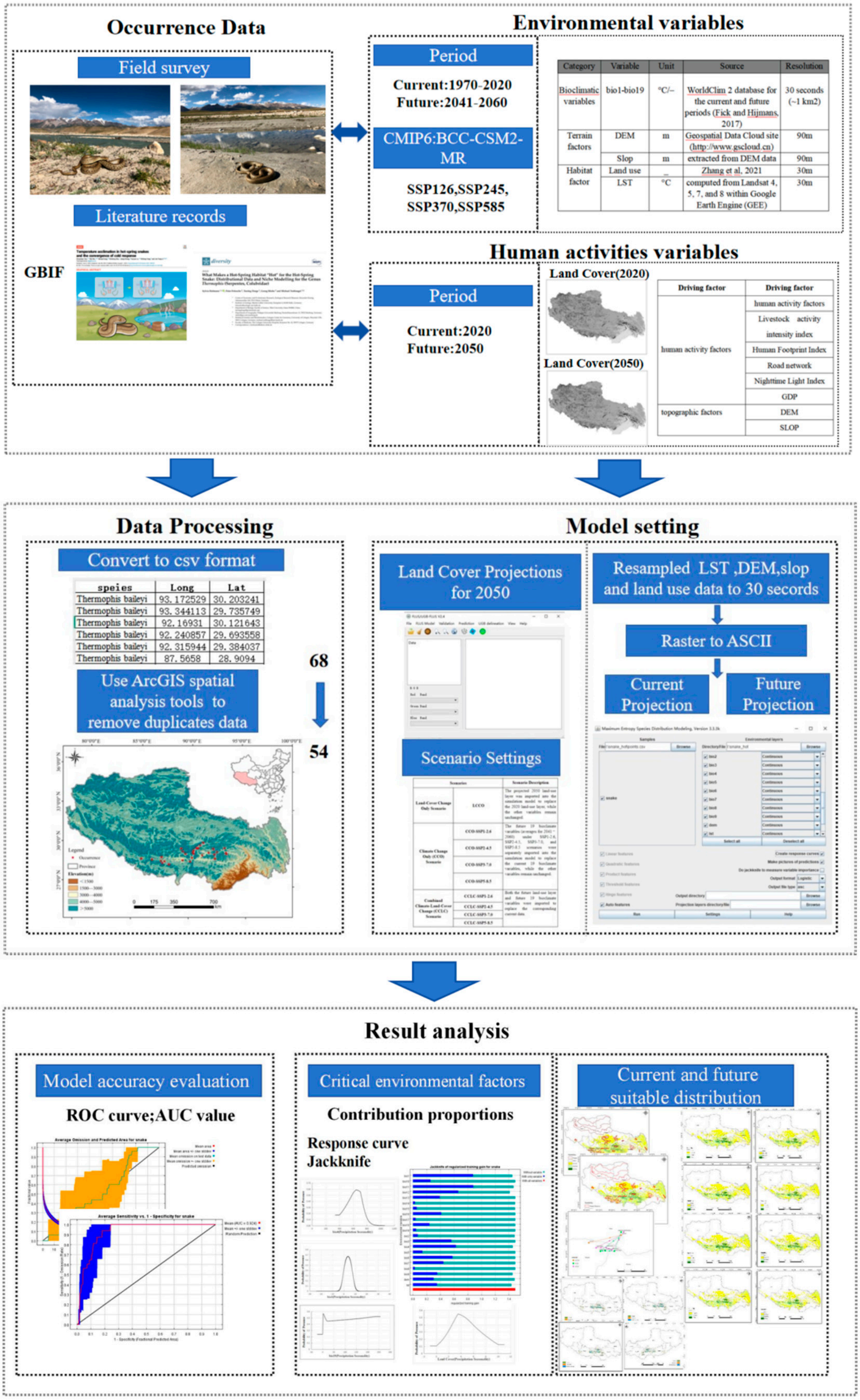

Figure S1. Research framework of this study.

Tables S1. 68 Occurrence records of *T. baileyi*

ID	Longitude	Latitude	Source	
1	93.341	29.740	field surveys	Reserve
2	93.180	30.200	field surveys	Reserve
3	92.280	29.692	field surveys	Reserve
4	92.140	30.153	field surveys	Reserve
5	92.144	30.140	field surveys	Reserve
6	92.290	29.363	field surveys	Reserve
7	90.351	29.980	field surveys	Reserve
8	90.350	29.978	field surveys	Remove
9	90.290	29.854	field surveys	Reserve
10	91.230	30.620	field surveys	Reserve
11	90.650	30.160	field surveys	Reserve
12	90.060	29.110	field surveys	Reserve
13	89.380	29.200	field surveys	Reserve
14	88.170	28.830	field surveys	Reserve
15	88.140	28.840	field surveys	Reserve
16	89.240	30.130	field surveys	Reserve
17	93.180	30.210	field surveys	Remove
18	92.270	29.690	field surveys	Reserve
19	92.290	29.354	field surveys	Reserve
20	90.350	29.980	field surveys	Reserve
21	90.290	29.850	field surveys	Remove
22	90.220	29.410	field surveys	Reserve
23	90.343	29.979	field surveys	Reserve
24	90.345	29.981	field surveys	Reserve
25	91.142	29.633	field surveys	Reserve
26	93.173	30.203	field surveys	Reserve
27	93.344	29.736	field surveys	Reserve
28	92.169	30.122	field surveys	Reserve
29	92.241	29.694	field surveys	Reserve
30	92.316	29.384	field surveys	Reserve
31	87.566	28.909	field surveys	Reserve
32	90.362	29.983	field surveys	Reserve
33	89.200	30.120	field surveys	Reserve
34	89.383	29.902	field surveys	Reserve
35	90.065	29.104	field surveys	Reserve
36	91.241	30.598	field surveys	Reserve
37	90.305	29.842	field surveys	Reserve
38	90.787	30.187	field surveys	Reserve
39	85.623	29.187	field surveys	Reserve
40	85.656	29.175	field surveys	Reserve

41	86.620	29.143	field surveys	Reserve
42	87.472	29.380	field surveys	Reserve
43	88.188	28.828	field surveys	Reserve
44	93.319	29.829	field surveys	Reserve
45	90.057	29.110	field surveys	Remove
46	90.220	29.420	field surveys	Remove
47	90.291	29.849	field surveys	Remove
48	90.306	29.840	field surveys	Remove
49	90.343	29.980	field surveys	Reserve
50	90.351	29.971	field surveys	Remove
51	90.361	29.980	field surveys	Remove
52	90.370	29.735	field surveys	Reserve
53	86.611	29.168	Yan et al, 2022	Reserve
54	87.450	29.406	Yan et al, 2022	Reserve
55	87.740	29.074	Yan et al, 2022	Reserve
56	87.428	29.090	Yan et al, 2022	Reserve
57	89.633	29.711	Yan et al, 2022	Reserve
58	90.371	29.734	Yan et al, 2022	Remove
59	90.593	30.200	Yan et al, 2022	Reserve
60	90.944	30.413	Yan et al, 2022	Reserve
61	92.488	29.896	Yan et al, 2022	Reserve
62	86.617	29.133	Hofmann et al, 2021	Reserve
63	87.453	29.407	Hofmann et al, 2021	Remove
64	87.465	28.910	Hofmann et al, 2021	Reserve
65	92.500	29.893	Hofmann et al, 2021	Reserve
66	91.100	29.600	GBIF	Remove
67	91.700	29.800	GBIF	Remove
68	90.300	30.010	GBIF	Remove

Tables S2. 54 Occurrence records of *T. baileyi*

ID	Longitude	Latitude	Source	Snakes occur
1	93.341	29.740	field surveys	occurrence
2	93.180	30.200	field surveys	occurrence
3	92.280	29.692	field surveys	occurrence
4	92.140	30.153	field surveys	occurrence
5	92.144	30.140	field surveys	occurrence
6	92.290	29.363	field surveys	occurrence
7	90.351	29.980	field surveys	occurrence
8	90.290	29.854	field surveys	occurrence
9	91.230	30.620	field surveys	occurrence
10	90.650	30.160	field surveys	occurrence
11	90.060	29.110	field surveys	occurrence
12	89.380	29.200	field surveys	occurrence

13	88.170	28.830	field surveys	occurrence
14	88.140	28.840	field surveys	occurrence
15	89.240	30.130	field surveys	occurrence
16	92.270	29.690	field surveys	occurrence
17	92.290	29.354	field surveys	occurrence
18	90.350	29.980	field surveys	occurrence
19	90.220	29.410	field surveys	occurrence
20	90.343	29.979	field surveys	occurrence
21	90.345	29.981	field surveys	occurrence
22	91.142	29.633	field surveys	occurrence
23	93.173	30.203	field surveys	occurrence
24	93.344	29.736	field surveys	occurrence
25	92.169	30.122	field surveys	occurrence
26	92.241	29.694	field surveys	occurrence
27	92.316	29.384	field surveys	occurrence
28	87.566	28.909	field surveys	occurrence
29	90.362	29.983	field surveys	occurrence
30	89.200	30.120	field surveys	occurrence
31	89.383	29.902	field surveys	occurrence
32	90.065	29.104	field surveys	occurrence
33	91.241	30.598	field surveys	occurrence
34	90.305	29.842	field surveys	occurrence
35	90.787	30.187	field surveys	occurrence
36	85.623	29.187	field surveys	occurrence
37	85.656	29.175	field surveys	occurrence
38	86.620	29.143	field surveys	occurrence
39	87.472	29.380	field surveys	occurrence
40	88.188	28.828	field surveys	occurrence
41	93.319	29.829	field surveys	occurrence
42	90.343	29.980	field surveys	occurrence
43	90.371	29.735	field surveys	occurrence
44	86.611	29.168	Yan et al, 2022	reference
45	87.450	29.407	Yan et al, 2022	reference
46	87.740	29.074	Yan et al, 2022	reference
47	87.428	29.090	Yan et al, 2022	reference
48	89.633	29.711	Yan et al, 2022	reference
49	90.593	30.200	Yan et al, 2022	reference
50	90.944	30.413	Yan et al, 2022	reference
51	92.488	29.896	Yan et al, 2022	reference
52	86.617	29.133	Hofmann et al, 2021	reference
53	87.465	28.910	Hofmann et al, 2021	reference
54	92.500	29.893	Hofmann et al, 2021	reference

Tables S3. Land-use reclassification

lc ID	35 land-cover sub-categories	re ID	10 land-cover sub-categories
10	Rainfed cropland	1	cropland
11	Herbaceous cover cropland	1	cropland
12	Tree or shrub cover (Orchard) cropland	2	forest
20	Irrigated cropland	1	cropland
51	Open evergreen broadleaved forest	2	forest
52	Closed evergreen broadleaved fores	2	forest
61	Open deciduous broadleaved forest (0.15<fc<0.4)	2	forest
62	Closed deciduous broadleaved forest (fc>0.4)	2	forest
71	Open evergreen needle-leaved forest (0.15< fc <0.4)	2	forest
72	Closed evergreen needle-leaved forest (fc >0.4)	2	forest
81	Open deciduous needle-leaved forest (0.15< fc <0.4)	2	forest
82	Closed deciduous needle-leaved forest (fc >0.4)	2	forest
91	Open mixed leaf forest (broadleaved and needle-leaved)	2	forest
92	Closed mixed leaf forest (broadleaved and needle-leaved)	2	forest
120	Shrubland	3	Shrubland
121	Evergreen shrubland	3	Shrubland
122	Deciduous shrubland	3	Shrubland
130	Grassland	4	Grassland
140	Lichens and mosses	4	Grassland
150	Sparse vegetation (fc<0.15)	3	Shrubland
152	Sparse shrubland (fc<0.15)	3	Shrubland
153	Sparse herbaceous (fc<0.15)	4	Grassland
181	Swamp	5	wetland
182	Marsh	5	wetland
183	Flooded flat	5	wetland
184	Saline	6	Saline
185	Mangrove	2	forest
186	Salt marsh	5	wetland
187	Tidal flat	6	Saline
190	Impervious surfaces	10	Urban land
200	Bare areas	7	Bare areas
201	Consolidated bare areas	7	Bare areas（Artificial solidification）
202	Unconsolidated bare areas	9	Bare areas（Exposed soil or rock areas）
210	Water body	5	wetland
220	Permanent ice and snow	8	glacier
0，250	Filled value		

Tables S4.Priority Conservation Area

Hot Spring Name	Location	Altitude(m)	Human Activities	Conservation Level	Protected Area	Habitat Quality Grade
Jiaduo Hot Spring	Jiaduo Village, Yangbajing Town, Damxung County	4400	Hot spring development, road construction	Priority Conservation	Conservation Gap	high suitability habitats
Qucan Hot Spring	Ranba Township, Renbu County	4098	Hot spring development, road construction	Priority Conservation	Conservation Gap	high suitability habitats
Kangma Hot Spring	Damxung County	4320	Hot spring development, road construction	Priority Conservation	Conservation Gap	high suitability habitats
Group 7 of Geda Township	Geda Township, Damxung County	4469	Hot spring development, road construction	Priority Conservation	Conservation Gap	high suitability habitats
Xumai Hot Spring	Xumai Township, Nyemo County, Shigatse	3928	Hot spring development, road construction	Priority Conservation	Conservation Gap	high suitability habitats

Material S1.

Questionnaire for the Tibetan Hot-spring Snake (*Thermophis baileyi*)

Basic Information

Site ID:`__________`

Date:`____/____/____` (YYYY/MM/DD)

Recorder:`__________`

Location: `__________`

Elevation: `__________ m`

Weather: ☀️ Sunny ☁️ Cloudy 🌧️ Rain ❄️ Snow 🌫️ Fog |Air Temp (°C):`____` |Ground Surface Temp (°C):`____`

A. Threats & Human Disturbance Assessment

1. Infrastructure & Development:

□ Geothermal Development (wells, plants, pipes) |Distance:`____ m` |Impact:□ Low □ Med □ High

□ Tourism Facilities (hotels, trails, platforms) |Distance:`____ m` |Impact:□ Low □ Med □ High

□ Roads |Distance:`____ m` |Traffic:□ Low □ Med □ High

□ Settlement/Pasture |Distance:`____ m`

2. Human Activity (observed on site):

□ Tourist throwing stones/playing □ Livestock trampling/drinking □ Bathing/laundry □ Trash pollution □ Intentional killing/harassment □ No obvious activity

3. Habitat Quality Perception:

Perceived habitat trend over the past ~5 years?

□ Significantly Improved □ Stable □ Slightly Degraded □ Severely Degraded □ Unknown

B. Conservation Status & Socioeconomic Information (From interviews with locals, herders, managers)

1. Land Ownership/Jurisdiction:

□ Nature Reserve □ National Park □ Community/Village Land □ Leased Enterprise

Land □ Other `__________`

2. Local Perception & Attitude :
a) Have you seen this snake? How often? (□ Common □ Occasional □ Rare)
b) Is it considered harmful? (□ Yes □ No □ Don't Know)
c) Is it considered valuable/useful? (Ecological, cultural, tourism?)
d) What is the biggest threat, in your opinion?
e) Do you support conserving this snake? (□ Strongly Support □ Support □ Neutral □ Oppose)

C. Overall Assessment & Notes

Initial Conservation Urgency for this site:
□Very High(Large population, severe & immediate threat)
□High(Stable population, but potential development threat)
□Medium(Small population, low disturbance)
□Low/Monitoring Needed(Stable population, no obvious threats)
